# Do females invest more into eggs when males sing more attractively? Postmating sexual selection strategies in a monogamous reed passerine

**DOI:** 10.1002/ece3.1034

**Published:** 2014-03-18

**Authors:** Ján Krištofík, Alžbeta Darolová, Juraj Majtan, Monika Okuliarová, Michal Zeman, Herbert Hoi

**Affiliations:** 1 Institute of Zoology, Slovak Academy of SciencesDúbravská cesta 9, Bratislava, 84506, Slovakia; 2 Department of Microbiology, Faculty of Medicine, Slovak Medical UniversityLimbová 12, 833 03, Bratislava, Slovakia; 3 Department of Animal Physiology and Ethology, Faculty of Natural Sciences, Comenius UniversityMlynská dolina, 842 15, Bratislava, Slovak Republic; 4 Department of Integrative Biology and Evolution, Konrad Lorenz Institute of Ethology, University of Veterinary Medicine ViennaSavoyenstr. 1a, Vienna, A – 1160, Austria

**Keywords:** *Acrocephalus scirpaceus*, egg yolk testosterone, lysozyme, male quality, song

## Abstract

Maternal investment can play an important role for offspring fitness, especially in birds, as females have to provide their eggs with all the necessary nutrients for the development of the embryo. It is known that this type of maternal investment can be influenced by the quality of the male partner. In this study, we first verify that male song is important in the mate choice of female Eurasian reed warblers, as males mate faster when their singing is more complex. Furthermore, female egg investment varies in relation to male song characteristics. Interestingly, clutch size, egg weight, or size, which can be considered as an high-cost investment, is not influenced by male song characteristics, whereas comparably low-cost investment types like investment into diverse egg components are adjusted to male song characteristics. In line with this, our results suggest that female allocation rules depend on investment type as well as song characteristics. For example, egg white lysozyme is positively correlated with male song complexity. In contrast, a negative correlation exists between-song speed and syllable repetitiveness and egg yolk weight as well as egg yolk testosterone concentration. Thus, our results suggest that female egg investment is related to male song performance in several aspects, but female investment patterns regarding various egg compounds are not simply correlated.

## Introduction

It is known that the phenotype of a mother, or the environment she experiences, can have an important effect on the phenotype and fitness of her offspring (Mousseau and Fox 1998). In fact, maternal investment may play a crucial role in modulating early developmental conditions, especially in birds, as females have to provide eggs with all the necessary nutrients for the development of the embryo before laying. Maternal investment may operate via behavior (e.g., start of egg laying, incubation, nestling feeding rates, or brood defense) or resource allocation toward eggs either via clutch size or the supply of egg compounds. Female egg investment may be influenced by the environmental and/or female condition (Moreno et al. 2006; Remeš 2011). Facultative and differential maternal investment has also been found as a function of male attractiveness (e.g., male display behavior and sexual ornaments) (Saino et al. 2002a,c2002c; Gil et al. 2004; Uller et al. 2005; Williamson et al. 2006; Loyau et al. 2007; Dentressangle et al. 2008).

In birds, male song is mainly driven by sexual selection (Andersson 1994) and has frequently been shown to be important in male–male competition and mate choice (Searcy and Yasukawa 1990). Females select males according to certain song characteristics such as song rate, song versatility, song types, or repertoire size (Buchanan and Catchpole 1997; Airey et al. 2000; Leitner and Catchpole 2007). All these song features are thought to be honest indicators of male quality (Zahavi 1975; Grafen 1990). In particular, song complexity offers multiple honest traits on which selection can act through female choice (Catchpole 1996; Gil and Gahr 2002) or maternal effects, whereby females may differentially allocate resources to eggs as a function of male song attractiveness. A few studies have already revealed that male song can influence female investment into clutch size or egg mass (Holvec and Riebel 2010) or testosterone concentrations in the egg yolk (Gil et al. 2004; Garcia-Fernandez et al. 2010). Investigating egg investment in relation to testosterone is particularly important, because an increase in egg yolk testosterone concentration can not only influence nestling begging behavior, and muscle development (Schwabl 1996; Lipar and Ketterson 2000), but may in turn influence the development of their offspring's sexual ornament, namely song quality, and in this way give females the opportunity to modulate the attractiveness of their male offspring. However, the only two experimental studies examining the effect of maternally derived testosterone on the development of their offspring song in canaries and starlings provided complex and nonsignificant results (Müller et al. 2008; Müller and Eens 2009). In the blood, circulating egg hormone concentrations, moreover, did not seem to pass to the egg yolk (Marshall et al. 2005). In terms of other egg compounds and the interrelationship between them, there is only limited evidence of song influencing female investment.

In this study, we therefore examine the female investment as a function of male song in the Eurasian reed warbler *Acrocephalus scirpaceus*, but female investment was investigated in terms of a series of egg properties and compounds including hormonal status (egg yolk testosterone, nutritional and antimicrobial substances, and structural egg features). To our knowledge, no study has investigated the role of male song in relation to female egg investment other than hormones, egg size, and mass, which could be seen as a possible indicator of nutritional resources provided to the egg. Our study is probably the first to investigate in detail the relationship between different male song parameters on the one hand and various egg investment parameters representing varying costs, including nutritional, antimicrobial, and hormonal substances provided to the egg as well as physical egg features.

The seasonally monogamous and monomorphic reed warbler is ideal for such an investigation, because male reed warblers' song is very complex. As in other *Acrocephalus* warbler species (Buchanan and Catchpole 2000), it has been suggested that their song is an indicator of male quality, constituting a sexually selected ornament involved in mate choice (Catchpole et al. 1984; Buchanan and Catchpole 1997; Marshall et al. 2007; see also this paper).

Furthermore, in an earlier study, we were able to demonstrate for the same species, that female quality itself is not a predictor for clutch size or egg quality, in terms of resources provided to the egg (Krištofík et al. 2013). That female adjustment in reproductive effort is independent of their own quality is known from several other species (Sheldon 2000). Thus, because egg investment is not obviously related to female quality, we, in a next step, want to determine how important male attractiveness might be to explain variation in egg investment.

Therefore, in this study, we determined the male song complexity of males and measured a variety of female egg investment parameters in terms of egg yolk testosterone concentrations, egg white lysozyme concentrations, nutritional parameters reflected by egg and egg yolk weight, and a structural egg feature (eggshell thickness).

Several predictions can be formulated in relation to male song quality and female mate choice and maternal investment: (i) Female reed warblers should prefer males whose song is more complex, (ii) female egg investment may also vary in relation to the song quality characteristics of the male mate, (iii) different male song characteristics may influence investment into different egg compounds, and (iv) female investment may vary in relation to the cost of the compound involved.

## Materials and Methods

### Study area

In the years 2009 and 2010, we studied reed warblers in the fishpond area of Veľké Blahovo, West Slovakia (48°03′09″N, 17°35′38″E). The area consisted of three fishponds covering an area of approximately 70 ha. The pond area is partly covered with marsh stands consisting of *Phragmites australis*,*Typha angustifolia*,*T. latifolia*, and partly *Carex* spp. In both study years, the entire area was inspected daily between 6:00 and 18:00 h, starting on 6 April until 10 July.

### Study species

The seasonally monogamous reed warbler is a long-distance migrant that overwinters in central Africa (Cramp 1992). At the beginning of April, reed warblers start to arrive in our study area. Immediately after their arrival, males start to sing a continuous and complex song that is very likely, as with many *Acrocephalus* warblers, an important feature of mate choice (see Results). Our population is mainly single brooded, but second broods occur exceptionally. Average clutch size is 4.0 ± 0.09 eggs (ranging from 3 to 5 eggs, *N *=* *42 clutches of 42 pairs). The average number of fledglings is: 2.7 ± 0.1 (range 1–5, *N *=* *42 broods of 42 pairs). In this study, we included data from 42 breeding pairs with a complete data set.

### Determination of male quality and female preferences

As a proxy for male intrinsic quality, we used arrival date (high-quality males may arrive earlier) (Smith and Moore 2005; Kipper et al. 2006; Tonra et al. 2011) and parameters related to song complexity (Kipper et al. 2006). Therefore, singing males were observed daily to determine male arrival in the territory. As a proxy for female preference, we used mating date (female arrival day at the territory) (Lozano 1994; Smith and Moore 2005) and time elapsed (number of days) between male and female arrival date. In order to determine the arrival day of the females (mating day), the territories were visited daily. A male was deemed to have attracted a mate when at least two of the following conditions were fulfilled: (i) The males reduced their song production (fewer and shorter strophes); (ii) the female was sighted in the territory; (iii) the male was sighted near the female; and (iv) the birds (male and female) gave alarm calls.

A few days after their arrival at the territory, male and female birds were mist-netted and individually color-ringed and four morphological (tarsus, wing, tail, and bill length) and one conditional (body mass) measurement were taken. To make sure that nothing else than male song performance influences female egg investment, we examined the possibility of assortative mating in morphology and condition. We found no assortative mating (correlation) in reed warbler pairs regarding tarsus, wing, tail, or bill length (Pearson correlation; for all *P *>* *0.3) or body mass and residual body mass not explained by size (Pearson correlation; for both *P *>* *0.7). To derive residual body mass, we used tarsus length as independent and body mass as the dependent variable. Thus, together with our earlier finding, namely that egg quality is independent of female quality (Krištofík et al. 2013), we can argue that female or male morphology or condition is unlikely to be very important for female egg investment.

Nests were checked daily in order to obtain information on reproductive parameters including the start of laying, clutch size, and the number of nestlings and fledglings.

Prior to mating, we collected song records from each male (approximately 5–10 min of song from different days). During the premating period, males sing throughout the whole day, but with a peak in the morning and the evening. The length of the premating period, however, varied a lot between different males, depending on mate acquisition. Therefore, for standardization and to reduce the possibility of variability in song recordings throughout the premating period, recordings were done only in the morning hours (between 6:00 and 10:00 h) and always on, or 1 day after, male arrival at the territory and with no neighbors singing or windy conditions. For 15 males, song was also recorded 4 days later (see below).

To analyze, 120 s of song takes about 2 days (depending on its complexity). As we had to balance our time in terms of quantity and quality, we decided to analyze all the males (recordings of 42 males) but for a shorter time period. For the analyses, we, therefore, randomly selected 30 s of song from the recorded song material of each male.

**Figure 1:**
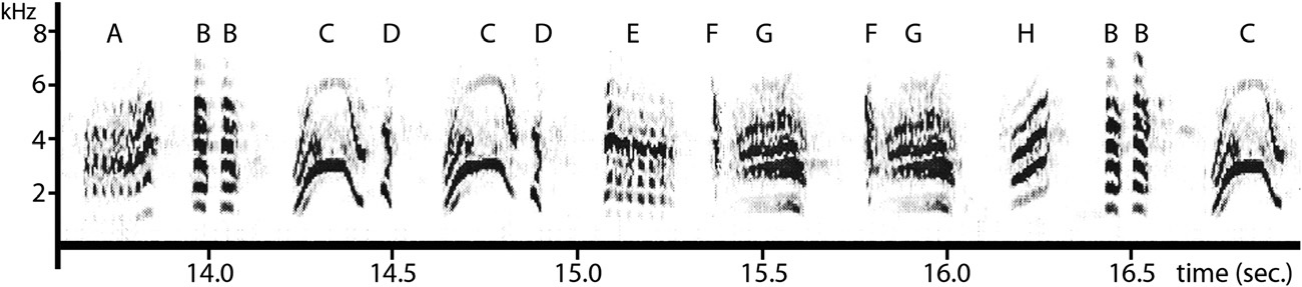
Sample (3.5 s) of male song. The smallest unit of each song are syllables of different structure which are separated by a short time of pause. In this sample, we can separate 16 syllables and eight of them are different.

For song recording, we used an M-Audiotrack digital recorder connected to a condenser microphone mounted on a Grampian parabolic reflector using a sampling rate of 44.1 kHz and a bit depth of 24 bit, an FFT length of 256 points on a flat-top window type for precise magnitude measurements, and in order to achieve a higher temporal resolution, a 50% overlap. For song analyses, we used Avisoft Bioacoustics software (Avisoft Bioacustics, Glienicke, Germany). The 30 s of song of each male was printed and subsequently analyzed. For the analyses, recordings were further divided into six-second intervals to determine structural parameters and the reliability of using 30 s of male song. This interval length was chosen based on early studies on marsh (*Acrocephalus palustris*) and moustached (*Acrocephalus melanopogon*) warblers with a similar complex song structure (for marsh warbler see Darolová et al. 2012; for moustached warbler see Fessl and Hoi 1996). Repeatability analyses (see Lessells and Boag 1987) revealed that using only six second of song could reliably characterize the song characteristics (complexity) of a male (see below), but to be on the safe side, we decided to analyze five times as much, namely 30 s of song. For each song, we determined syllables (for a definition of syllables Catchpole and Slater 1995) as the smallest structural unit. As exemplified in Figure 1, different syllables have a different structure and are separated by a short pause. They are determined and separated by eye. To reduce errors due to variation in syllable discrimination, only one person (AD) did the analyses, using exactly the same criteria and precision for all males.

The song of male reed warblers is very complex, for example, the average number of syllables/minute calculated for 15 adult reed warblers is 360, but varies between males from 244 up to 434 syllables. The average number of different syllables/minute a male produces is 144 and varies between 102 and 218 different syllables (*N *=* *15 males). Typically for reed warblers, it is not only the high structural complexity, even in comparison with other *Acrocephalus* warblers (for a review see Leisler and Schulze-Hagen 2011), but also that males frequently repeat syllables several times before they switch to another, that is, new syllable type. Our own observations suggest that actually song repetitiveness seems to be heritable. Furthermore, the high variation in the number of syllables produced per time unit also suggests that song speed varies significantly among male reed warblers. Thus, reed warbler song is not just a matter of complexity, but a multiple ornament where probably a combination of different characteristics related to syllable complexity, repetitiveness, and song speed might be involved in female choice and maternal investment rules. We do not know which song characteristics may best explain song structure and which might be important in mate choice. Therefore, to compare the song structure of adult male reed warblers, we determined eight different song variables describing song speed (variable 1), song complexity (variables 2–4 and 8) and simplicity, and repetitiveness (variables 5–7; see also the example in Table 1). To evaluate whether the analyzed song period (30 s) is sufficient to describe the song of a male based on the eight variables, we first calculated the within-song recording repeatability of all parameters. Therefore, we determined the repeatability of each variable based on six-second intervals for the 30 s. We would expect that a male whose song is more complex should do so at any of the six-second intervals within the 30 s of song. In fact, we found significant repeatability in all eight parameters investigated (see below). Furthermore, we calculated the repeatability between-song recordings of all eight parameters for each six-second interval (30 s) for two recordings from different days (day of arrival and 4 days later), which were analyzed for 15 males. Again, we found a high repeatability between the two song recordings from the same male. The following eight variables have been investigated:

1. Song speed: This parameter describes song speed in terms of the number of song syllables a male produces. The number of syllables/30 s was used in the analyses. Based on song speed of six-second intervals, there is high within-song (*r *=* *0.74, *F*_40,205_* *=* *9.93, *P *<* *0.0001) and between-song recording repeatability (two recordings from different days: *r *=* *0.79, *F*_13,140_* *=* *10.8, *P *<* *0.0001).
2. Number of syllable types: This variable refers to structural complexity, which takes versatility but not necessarily innovative skills into account. It describes how many different syllable types a male produces in six-second intervals independent of whether the same syllables are used in other intervals. The total number of syllable types of five–six-second intervals (30 s) is used for the analyses. Again, we found significant within-song (*r* = 0.61, *F*_40,205_* *=* *5.13, *P *<* *0.001) and between-song recording repeatability (*r *=* *0.59, *F*_13,70_* *=* *4.85, *P *<* *0.001).
3. Number of new syllables: This complexity variable is a mixture between an innovative and a frequency aspect and counts the appearance and frequency of new syllable types at every six-second interval. The total number of new syllables pooled over four intervals (30 s) is used for the analyses. Within-song (*r *=* *0.75, *F*_40,164_ = 8.41, *P *<* *0.0001) and between-song recording repeatability (*r *=* *0.72, *F*_13,70_* *=* *7.8, *P *<* *0.0001) are significant.
4. Number of new syllable types: This variable describes complexity in relation to the degree of innovation. It describes the number of new types of syllables appearing every six second during 30 s of a male's song. The total number of new syllable types appearing over four intervals (30 s) is used for the analyses. Assuming high consistency in this variable over time, this variable would be the best predictor for repertoire size. Within-song (*r *=* *0.89, *F*_40,164_* *=* *15.3, *P *<* *0.0001) and between-song recording repeatability (*r *=* *0.88, *F*_13,112_* *=* *15.1, *P *<* *0.0001) are highly significant.
5. Number of repeated syllables: This variable describes simply how frequently a syllable type appears in a six-second interval. The total number of syllable repetitions over five intervals (30 s) is used for the analyses. There is within-song (*r *=* *0.54, *F*_40,205_* *=* *3.3, *P *<* *0.01) and between-song recording repeatability (*r *=* *0.51, *F*_13,140_ = 3.1, *P *<* *0.01).
6. Number of repeated syllable types: This variable describes how many different syllable types are repeatedly advertized per six-second interval. The total number of syllable repetitions over five intervals (30 s) is used for the analyses. Within-song (*r *=* *0.89, *F*_40,205_* *=* *15.1, *P* < 0.0001) and between-song recording repeatability (*r* = 0.88, *F*_13,140_* *=* *14.8, *P *<* *0.0001) are highly significant.
7. Number of immediately repeated syllables: This variable describes the number of syllables that are immediately repeated syllables over 30 s based on six-second intervals. The total number of new syllable types appearing over five intervals (30 s) is used for the analyses. There is also high within-song (*r *=* *0.92, *F*_40,205_* *=* *16.1, *P *<* *0.0001) and between-song recording repeatability (*r *=* *0.86, *F*_13,70_* *=* *14.4, *P *<* *0.0001).
8. Number of syllable switches: This complexity variable counts how frequently a male switches to a different syllable between successively produced syllables. Counts are again based on six-second intervals. The total number of syllable switches appearing over five intervals (30 s) is used for the analyses. Within-song (*r *=* *0.56, *F*_40,205_* *=* *3.83, *P *<* *0.01) and between-song recording repeatability (*r *=* *0.51, *F*_46,140_* *=* *3.79, *P *<* *0.01) are significant.

**Table 1:** Examples of different song parameters. The table (i) exemplifies how the different song parameters have been calculated, based on a sequence of different syllables (second row e.g., A, B, C, etc.) appearing during different time spans. During the first (0–6 s), and the second (7–12 s), six-second interval of 30 s of song. The numbers resulting for each song parameter are given in the two columns, respectively. Results are shown for the eight song parameters investigated. The number of new syllables and syllable types can only be determined from the second interval onward.

Time span	0–6 s	7–12 s
Sequence of syllables	AABCDEEA	EBBFGGGEH
Syllables (speed)	8	9
Syllable types	5	5
New syllables	–	5
New syllable types	–	3
Repeated syllables	5	7
Repeated syllable types	2	3
Immediately repeated syllables	4	5
Syllable switches	5	5

### Egg quality measurements

The egg quality of the third egg from 80 clutches was determined, but in this study, we use only the third egg from 42 clutches from which we have the complete data set (see earlier). To be able to collect the third egg from each clutch, pairs were observed daily to find the nest and to determine the start of egg laying. As the females lay one egg a day, we marked the first and second laid egg with a little point on the shell, in order to distinguish the third egg from the first two eggs. The third egg was then removed from each nest and replaced by a dummy egg made of epoxide. No brood was deserted because of our manipulation. The removed egg was immediately put into a cooling box (4°C) for transportation and, after arrival in the lab, was frozen at −80°C.

To determine egg quality, we measured (i) egg size, (ii) eggshell thickness, (iii) egg weight, and (iv) yolk weight and estimated (v) antimicrobial egg white lysozyme activity, and (vi) egg yolk testosterone concentration (T) for each egg.

1. Egg size (egg length and maximal width) was measured with an electronic caliper to the nearest 0.01 mm immediately after egg removal.
2. Eggshell thickness was measured using a micrometer (Somet) to the nearest 0.001 mm. For each egg, we measured eggshell thickness on five different points at the central egg area. For the analyses, we used the average thickness of these five points.
3. Fresh egg weight was determined with an electronic balance to the nearest 0.0001 g, about 3 (10) h after sampling.
4. Eggs have been frozen for about 1 month. For the analyses, eggs were defrosted and egg yolks were carefully dissected from thawing albumen. Frozen yolks were weighed, and both egg white and yolk were than stored at −80°C until lysozyme and testosterone analyses. Eggshells were rinsed under warm distilled water and dried at room temperature for later measurements of eggshell thickness.
5. Lysozyme analyses: Lysozyme is the most well-known antimicrobial protein in egg white. Lysozyme is effective against Gram-positive bacteria, but it shows little to no antibacterial effect against Gram-negative bacteria. Hen egg is the richest source of lysozyme accounting for 3.5% of total egg white proteins (Charter and Lagarde 1999). To determine the antimicrobial activity of lysozyme, we prepared the albumen samples, which were collected into preweighed 2.0-mL reagent tubes. Each tube with an albumen sample was weighed, and the albumen was lyophilized. Lyophilized powder was subsequently dissolved in 200 *μ*L of distilled water, and the obtained albumen solution was used to determine antibacterial activity. Radial diffusion assay was used in order to evaluate the lysozyme concentration of albumen samples. Briefly, one bacterial colony of overnight agar plate culture of *Micrococcus luteus* was suspended in phosphate-buffered saline (PBS), and the turbidity of suspension was adjusted to 10^8^ CFU/mL. A 100-*μ*L aliquot of suspension was inoculated to 10 mL of melted LB broth (Luria broth) containing 0.9% (w/v) agar preheated at 48°C and poured into 90-mm Petri dishes. After solidification, 5-mm diameter wells were punched into LB agar, and 5 *μ*L of sample was added to each well. Antibacterial activity of examined samples was compared on the basis of radius of clear inhibition zone around the well against standard solutions of a chicken egg white lysozyme (Sigma-Aldrich, Munich, Germany) after 24 hours of incubation at 37°C. Antibacterial activity of albumen samples was expressed as concentration of egg white lysozyme (*μ*g/g of egg white) (Majtan et al. 2012). The results given represent mean values from duplicate measurements of each independent sample.
6. Testosterone analyses: Yolk T concentrations were determined by radioimmunoassay after yolk steroid extraction (Okuliarová et al. 2010). Subsamples of 40–45 mg of yolk were diluted in 500 *μ*L of deionized water and vortexed with the addition of two glass beads for three minutes. Approximately, 1500–2000 dpm of [^3^H]-testosterone were added to each sample for individual recovery calculation (mean ± SE: 55.0 ± 0.8%). Samples were equilibrated overnight at 4°C. Thereafter, they were applied on solid phase columns filled with Extrelut NT (Merck, Darmstadt, Germany) and extracted with 2 × 2 and 1 × 1 mL of a mixture of diethyl ether and petroleum ether (7:3). Following evaporation under a stream of nitrogen, the dried extracts were reconstituted in 300 *μ*L of phosphate buffer (pH* *=* *7.5) and frozen until T assay. Yolk T concentrations were measured in 10-*μ*L aliquots of the extract using [1,2,6,7-^3^H]-testosterone (Amersham Biosciences, Buckinghamshire, U.K., specific activity 3.52 TBq/mmol) and a specific rabbit antibody against testosterone-3-(carboxy-methyl) oxime-bovine serum albumin conjugate. Cross reactivity of antiserum was 9.6% with 5*α*-dihydrotestosterone and lower than 0.1% with other steroids (Zeman et al. 1986). All samples within each year (2009 and 2010) were run in a single assay with intraassay variation coefficients of 7.6% and 2.2%, respectively.

Finally, also clutch size was used as a parameter for costly female investment in relation to male attractiveness.

Trapping, measurement of adult reed warblers and removing one egg from each nest were carried out in accordance with the valid legislation and were approved by the Ministry of the Environment of the Slovak republic under the number 3513/2009-2.1/jam.

### Statistical analyses

The study was conducted over 2 years, but each pair was entered only once in the data set. Before examining the relationship between male song parameters and female investment, we used a principal component analysis to reduce the number of song variables to a subset of unrelated song factors. A larger number of explanatory variables always provide a greater chance of overfitting the model, particularly when there are strong intercorrelations between different possible explanatory variables, which can be expected for the eight song variables investigated. As a result of the PCA, we obtained two independent significant principal components (PC I and PC II) explaining 71% of the total variance. PC I explains 39%, and all parameters with high factor loadings with PC I are consistently descriptors of song complexity. Thus, PC I can be very clearly termed as a song complexity factor (see Table 2). PC II explains 32%, and in contrast, all variables describing syllable repetitions show high factor loadings on PC II, which makes it reasonable to label this factor a repetition factor. However, additionally, the high factor loading of song speed on PC II means that increasing song speed is mediated by increasing syllable repetitions. However, the increasing speed may by default to a smaller extent and also drive the appearance of new syllables, which is suggested by the moderate factor loading of this variable (see Table 2). In conclusion, PC II can be seen as a syllable repetition and song speed factor (see Table 3). For the later analyses, we used the factor scores of each male on each principal component. To examine whether males arrive earlier, or sing in a more complex way, a stepwise regression analysis was used. Therefore, male arrival date was used as dependent variable and the two song quality factors as independent variables. Arrival date was expressed as 1 April being day 1. To examine whether male song quality (songs PC I and II) influences female arrival at the territory and mating speed, we used stepwise regression analysis with a) female arrival date (1 April is day 1) and b) female mating speed (days elapsed between male and female arrival) as dependent variable. The two male song quality factors (PC I and PC II) and for mating speed also the date from the start of egg laying (to control for season) were introduced as independent variables.

**Table 2:** Results of a principal component analyses resulting in two principal components (PC I and PC II). Given are factor loadings (>0.5) of each song variable (see Table 1) to PC I and PC II and the variance explained (%).

Factor	PC I	PC II
Number of syllables (speed)	0.2	−0.87
Number of syllable types	0.87	−0.07
Number of new syllables	0.52	−0.53
Number of new syllable types	0.89	0.10
Number of repeated syllables	−0.22	−0.85
Number of repeated syllable types	0.13	−0.62
Number of immediately repeated syllables	−0.59	−0.66
Syllable switches	0.84	0.09
Variance explained	39	32

**Table 3:** Results of the multiple stepwise regression model for each male attractiveness factor. Song component I: indicator for song complexity and song component II: indicator for syllable repetitions and song speed. Given are the partial regression coefficients and in parenthesis the respective *P*-values. Significant results are bold. For the complete regression model statistics regarding significant parameters see Results section.

	Song component I Complexity	Song component II Repetitions and speed
Clutch size	0.06 (>0.8)	−0.15 (>0.3)
Chick number	0.03 (>0.8)	−0.11 (>0.5)
Egg size	0.09 (>0.7)	−0.23 (>0.2)
Shell thickness	0.001 (>0.9)	0.009 (>0.9)
Lysozyme	**0.52 (=0.009)**	−0.20 (>0.2)
Testosterone	−0.009 (>0.9)	**+0.44 (=0.026)**
Egg yolk weight	−0.007 (>0.9)	**−0.40 (=0.021)**

Furthermore, stepwise regression analysis was used to examine which egg quality and which male quality parameter are related. Regression models were performed separately for each female investment parameter as the dependent variable and the two male song factors and year as independent variables. For statistical analyses, we used IBM SPSS Statistics 20 (IBM Corp., Armonk, NY).

## Results

### Male quality features and female preferences

**Figure 2:**
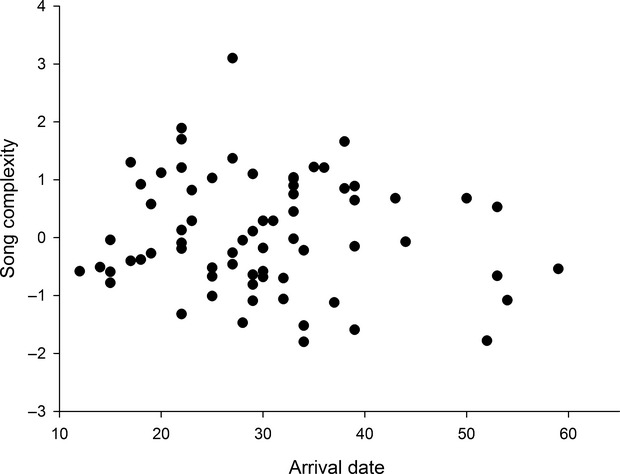
Relationship between male arrival date and song complexity (song factor I). Arrival date is expressed as days after the 1th April.

We can show that arrival at the breeding ground is related to song features. Song complexity significantly entered a stepwise regression model (*F *=* *8.3, *P *=* *0.009, *R*^*2*^* *=* *0.29, df* *=* *1.41) using arrival date as the dependent variable. The partial regression coefficient reveals that males arriving earlier sing in a more complex way (*r*_part_* *= −0.54; Fig. 2).

Regarding mating date, again song complexity entered the stepwise regression model (*F *=* *4.6, *P *=* *0.016, *R*^*2*^* *=* *0.17, df* *=* *1.41). Males getting a female earlier sing more complexly (*r*_part_ = −0.41), and more important males getting mated faster (controlling for female first egg) also seem related to male song PC I (stepwise regression model: *F *=* *23.5, *P *<* *0.0001, *R*^*2*^ = 0.43, df* *= 2.41, *r*_part_ = −0.66).

### Female investment and male quality

Examining clutch size, egg weight and size, and shell thickness in relation to the two song factors, no variable entered the regression model (*P *>* *0.2).

**Figure 3:**
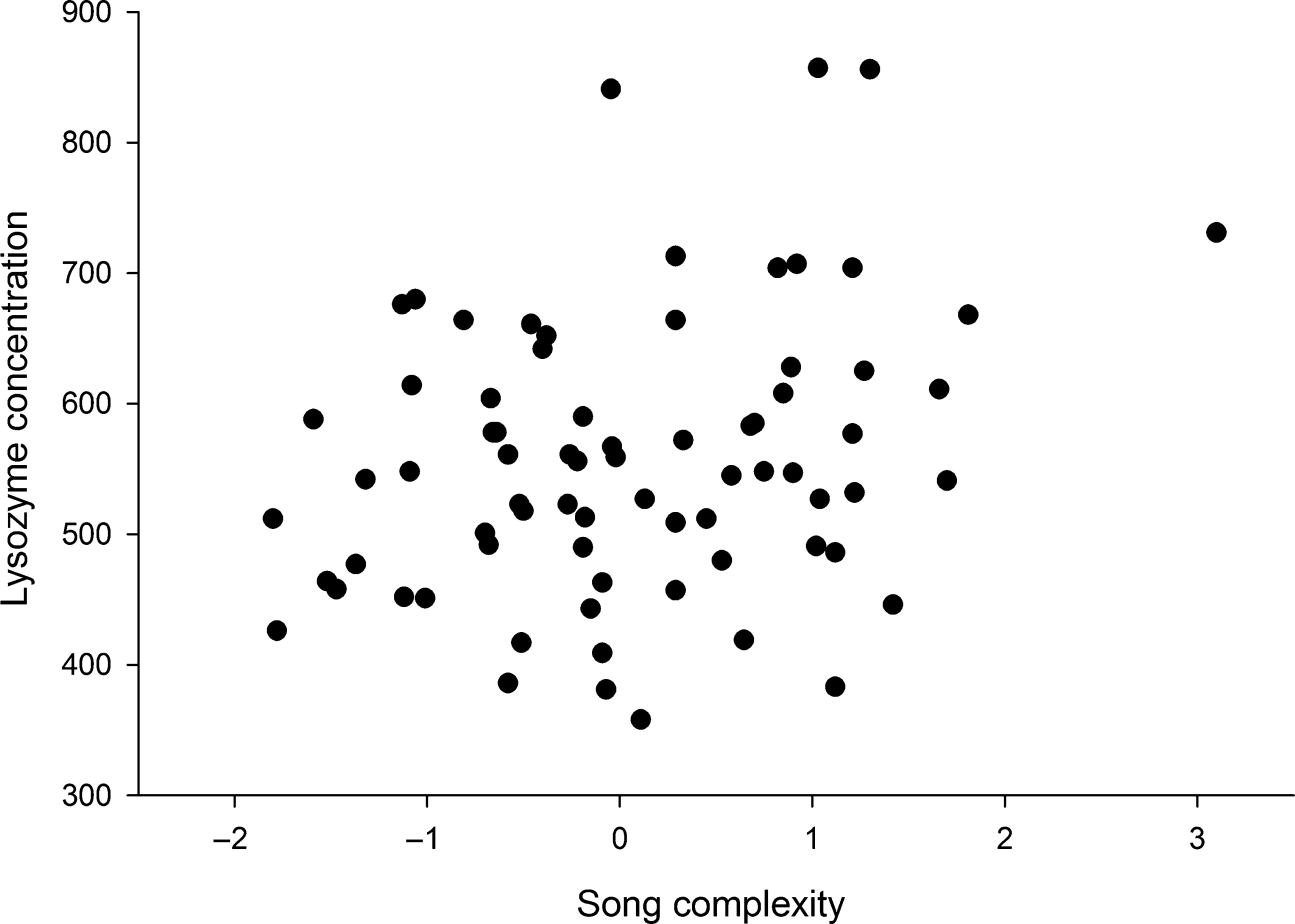
Relationship between-song factor I and lysozyme concentration (*μ*g/g of egg white).

Examining egg content, we found a significant correlation between egg white lysozyme and song complexity (*F *=* *8.2, *P *=* *0.009, *R*^*2*^ = 0.27; df = 1.41 for song complexity factor: *r*_part_ = 0.52; Fig. 3). This suggests that females mated to males producing more complex song put more lysozyme into their eggs.

**Figure 4:**
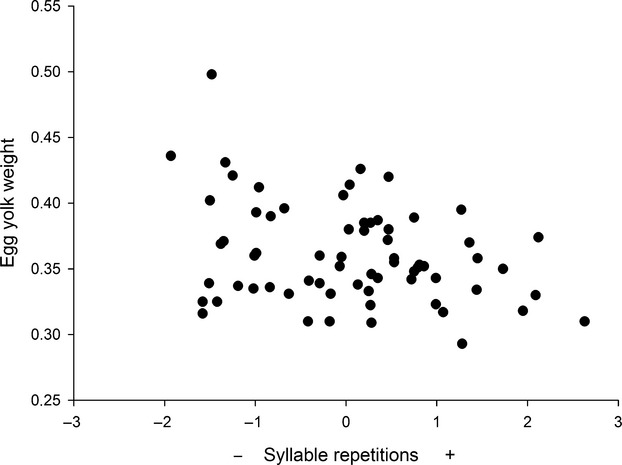
Relationship between-song factor II and egg yolk weight.

We found a negative correlation between egg yolk weight and song (*F *=* *5.31, *P *=* *0.026, *R*^*2*^* *=* *0.17, df* *= 1.41 for syllable repetition and song speed factor: *r*_part_ = −0.39; Fig. 4), which suggests that females produce less egg yolk when their males sing faster and repeat syllables more frequently.

Finally, we found a correlation between egg yolk testosterone concentration and male song (*F *=* *4.51, *P *=* *0.021, *R*^*2*^* *= 0.21, df* *= 1.41 with song factor II*: r*_part_ = 0.44). Thus, females mated to males that sing faster and repeat syllables more frequently have relatively more testosterone in their eggs. However, this relationship disappears if we use total egg yolk testosterone instead of testosterone concentration. No song component entered the analyses (original model, *F *=* *0.21, *P > *0.8, *R*^*2*^ = 0.004, df* *= 3.41).

The number of chicks fledged is independent of male song quality features. No variable entered the model (*P *>* *0.4).

## Discussion

In this study, we found evidence in reed warblers that male song is a quality indicator. Arrival time is frequently mentioned as a quality indicator, with males that arrive earlier being in better condition and heavier (Kipper et al. 2006); they also have higher androgen levels (Tonra et al. 2011), start breeding earlier (Smith and Moore 2005), and have larger repertoires (Kipper et al. 2006) and better territory (Lozano 1994). In line with this, we found that male reed warblers that arrive earlier sing with more complexity than males arriving later. Furthermore, we were able to show that, independent of the time of season, song complexity influenced male mating success. Males that sing more complexly mate faster, than males with less complex songs.

The function of song complexity in mate choice is not surprising. There is evidence from several other species, in particular *Acrocephalus* warblers that song complexity, usually in terms of repertoire size, is involved in mate choice (Catchpole 1996; Buchanan and Catchpole 1997), and reviewed in Byers and Kroodsma (2009). Female preferences for larger song repertoires and more complex songs seem likely to be adaptive, because song complexity is known to positively correlate with a number of traits important to fitness, such as territory tenure, heterozygosity, immune system quality, longevity, and lifetime reproductive success (Hiebert et al. 1989; Reid et al. 2005a,b2005b; Pfaff et al. 2007). Females that prefer males with higher song complexity (quality) may also obtain direct benefits, as these males may also be more proficient in other cognitively demanding behaviors, such as learning when, where and how to feed (Nowicki et al. 2000; DeVoogd 2004), and how to cope with environmental changes (Botero et al. 2009). However, female response in relation to male quality can also be examined at the postmating stage. Several studies have revealed variation in female investment into reproduction according to male attractiveness, for example, clutch size (Petrie and Williams 1993; Mousseau and Fox 1998) or feeding effort (Sheldon 2000). Several studies also found an effect of male attractiveness on egg features, for example, egg volume (Uller et al. 2005; Mcfarlane et al. 2010), egg mass (Cunningham and Russell 2000; Gilbert et al. 2006; Bonato et al. 2009; Giraudeau et al. 2011), egg size (Horváthová et al. 2012), yolk mass (Remeš 2011), testosterone concentration (Gil et al. 2006; Loyau et al. 2007; Safran et al. 2008; Garcia-Fernandez et al. 2010; Loyau and Lacroix 2010), lysozyme (D'Alba et al. 2010; Giraudeau et al. 2011), carotenoids (Saino et al. 2002c; Hargitai et al. 2008), and corticosterone (Pike and Petrie 2005). However, only a few studies we are aware of, found a relationship between-song characteristics and egg investment, mainly in relation to clutch size, egg, or egg yolk volume (Holvec and Riebel 2010; Garcia-Fernandez et al. 2013; Okanoya and Soma 2013) or some in egg compounds, in particular egg yolk testosterone (Gil et al. 2004; Garcia-Fernandez et al. 2010). Half of these studies have been looking on song production rather than song structure.

When examining male attractiveness in relation to mate choice, many studies focus on a single ornament, usually a morphological and sexual dimorphic trait, which varies, for example, in size or coloration (Gil et al. 2006; Dentressangle et al. 2008; Hargitai et al. 2008; Mcfarlane et al. 2010). It is becoming clearer that mate choice is based on multiple rather than a single trait (Griggio et al. 2011). Therefore, female allocation rules regarding different egg compounds might also be more complex in the way female investment may be adjusted to different male traits. In the same way, when examining female investment into an egg, studies usually concentrate on only one or a few egg compounds at the same time (Gil et al. 2007). Females, however, transfer many substances to their eggs, and these egg substances have different functions. Egg substances are not likely to act independently, but compounds may also interact, that is, compensate for others (Vallarino et al. 2012). Thus, an investigation of single parameters might give only limited insight into the whole picture. Also, the relation between female investment and male attractiveness is not clearly defined. Females may invest more into nutritional egg compounds when mated to low-quality, less attractive males for compensatory reasons (Gowaty 2008; Bolund et al. 2009). Contradictory results have also been found regarding egg yolk androgens (Gil et al. 2006; Dentressangle et al. 2008; Ratikainen and Kokko 2010).

Here, we investigate simultaneously different potential male quality parameters related to song and their relationship to female investment into a number of egg characteristics and compounds. There is no obvious sex difference in coloration or other ornament, and plumage is dull brown; hence, we assume that coloration is not important for mate choice in this species. Furthermore, female adjustment into reproduction is often independent of her own quality (Sheldon 2000). A direct female quality effect on egg investment can also be neglected since in an earlier study, we did not find a relationship between female quality (morphology and condition) and clutch size or any investment into the egg (Krištofík et al. 2013).

Our results show that female investment into reproduction is influenced by the obvious male ornament, namely elaborate male song. In fact, song complexity turned out to be a good predictor for various egg compounds, for example, lysozyme, testosterone, and egg yolk weight, but interestingly, the direction of the relationship varies for the different egg compounds.

Male song predicts the lysozyme and testosterone concentration of an egg. Both lysozyme and testosterone production and deposition have been shown to be costly for females [for lysozyme (Saino et al. 2002b, 2007), for testosterone (Gil et al. 2006)]. We found that females mated to males that sing more complexly (have high factor scores on song factor I) have higher concentrations of egg white lysozyme. A relation between lysozyme concentration and male ornaments, in particular coloration, was also found in some other studies (D′Alba et al. 2010; Giraudeau et al. 2011). Lysozyme is important for the embryo because this antimicrobial substance may be carried over from albumen into the nestling for a short period after hatching and may influence its health status and survival (Saino et al. 2002b).

Regarding egg yolk testosterone concentration, we found a positive correlation with syllable repetitions and song speed (PC II). Thus, when females are mated to males that repeat syllables more frequently and sing faster, their eggs contain a higher concentration of yolk testosterone. However, total yolk testosterone is not related to male song, which suggests that regarding testosterone, relative but not absolute female investment varies.

As evidenced, females mated to males that sing more complex songs invest more into egg lysozyme, whereas females that are mated to males that repeat syllables more frequently and sing faster have higher testosterone concentrations. This result does not mean that there is a trade-off between testosterone and lysozyme. However, song complexity and syllable repetitions are correlated with two independent principal component factors. Thus, males that repeat syllables more frequently and sing faster do not necessarily sing with less complexity. Song complexity and syllable repetitions with song speed are rather two independent descriptors of male song in reed warblers that could be independently used in mate choice. In fact, a direct correlation between lysozyme and testosterone revealed no relationship at all (*P *>* *0.8). Consequently, the independence of song complexity and syllable repetitions means that an increase in song complexity mediated in lysozyme does not necessarily mean a decrease in syllable repetitions mediated by testosterone.

Furthermore, male song is also related to egg yolk weight, but in contrast to testosterone, the relationship between egg yolk weight and the syllable repetitions factor was negative, which suggests that females provide less nutrition to eggs when their males repeat the same syllables more frequently.

Thus, in male reed warblers, song seems to have a great influence by stimulating female reproduction whereby female physiological responsiveness may depend on the degree of song – complexity as well as simplicity. However, female reproductive investment is not always directly related to the one and most conspicuous trait related to male attractiveness. Female investment might be more subtle and include other less obvious traits. Female collard flycatchers, for example, do not adjust their reproductive investment to the most obvious trait related to male attractiveness but to male age (Michl et al. 2005).

In conclusion, female choice and postmating investment in reproduction are more complex even when only one conspicuous ornament is involved. Female investment regarding various egg compounds is not simply correlated but may differ according to the function or need, and the responsiveness for each egg compound may depend on various pieces of information collected from males, and the environment, and may also depend on her own condition.
